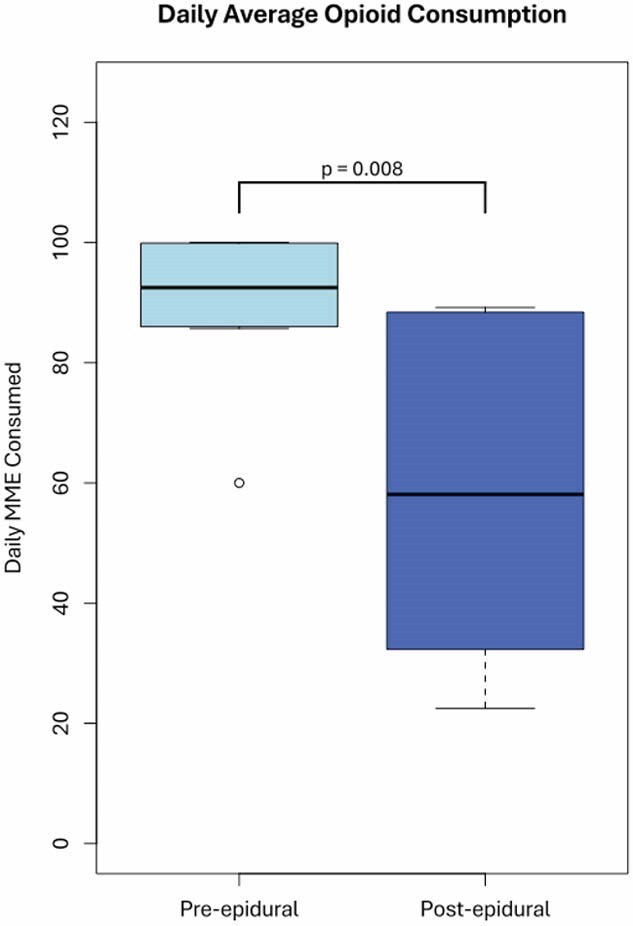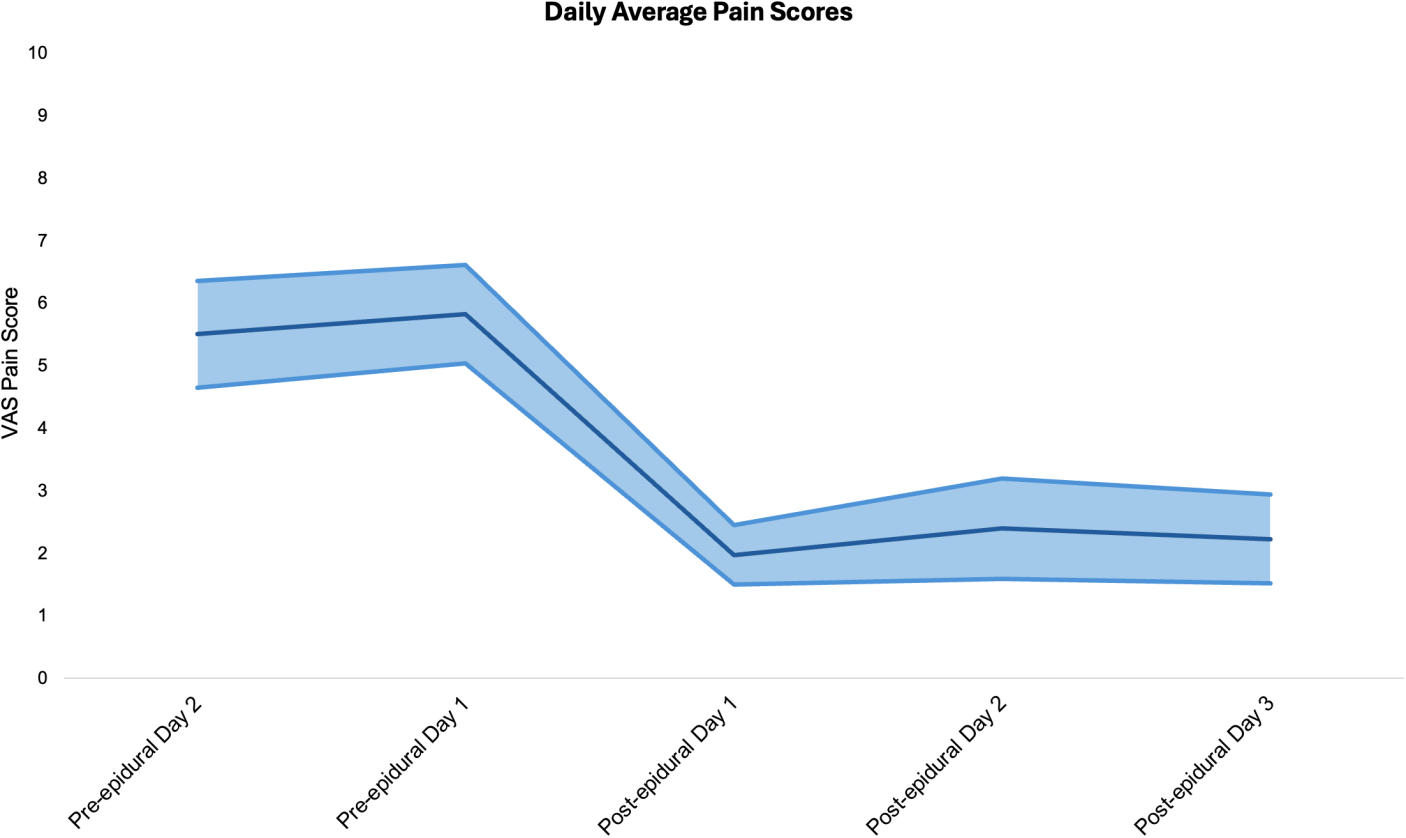# 928 Epidural Anesthesia for Pain Relief in Patients with Severe Burns

**DOI:** 10.1093/jbcr/iraf019.459

**Published:** 2025-04-01

**Authors:** Max Silverstein, Ujalashah Dhanani, Pandora Chua, Clifford Sheckter, Yvonne Karanas

**Affiliations:** Division of Plastic and Reconstructive Surgery, Stanford University; Department of Anesthesiology, Santa Clara Valley Medical Center; Department of Anesthesiology, Santa Clara Valley Medical Center; Santa Clara Valley Medical Center; Santa Clara Valley Medical Center

## Abstract

**Introduction:**

Patients with severe burns endure intense pain, which is amplified by serial operations, daily dressing changes, and regular physical/occupational therapy. Inadequate pain control causes secondary psychological trauma and opioid dependence. Regional anesthesia techniques are effective for managing pain in a variety of clinical scenarios, including severe burns. While peripheral nerve blocks have become increasingly popular in the management of isolated burns to the extremities, there have been few reports on the use of neuraxial anesthesia to treat burn pain. Here, we describe the inclusion of epidural anesthesia in our algorithm for management of burns to the lower trunk, bilateral lower extremities, buttocks, and perineum. We hypothesized that epidural anesthesia would be associated with reduced opioid use and improved pain scores.

**Methods:**

We performed a retrospective review of all patients admitted to a verified burn center who underwent epidural catheter placement between 2018 and 2024. Visual analog scale (VAS) pain scores and opioid consumption (standardized in morphine milligram equivalents [MME]) were extracted for the several days before and after placement of each patient’s first epidural catheter. Statistical testing was performed with the Wilcoxon Rank Sum Test (significance at p ≤ 0.05).

**Results:**

11 patients underwent epidural catheterization in our burn unit. Of those patients, 4 were male and 7 were female, with a mean age of 54.8 (SD 22.0, range 12 – 88) years. An average of 1.8 (SD 1.3) catheters were placed per patient, for a total of 20 catheters. Epidural catheters were removed 4.0 (SD 1.9, range 1 – 8) days after placement. All patients experienced significant reductions in daily opioid consumption (92.5 MME/day pre-epidural to 58.1 MME/day post-epidural, p = 0.008) and average pain scores (6.25 pre-epidural to 2.45 post-epidural; p = 0.008). Minor complications including nausea/vomiting and pruritis occurred in 4 patients. There were no major complications or infections.

**Conclusions:**

Epidural anesthesia is safe and effective for relieving pain and decreasing opioid consumption in burn patients.

**Applicability of Research to Practice:**

Burn centers should consider neuraxial anesthesia for pain management in appropriate patients. These techniques require close collaboration between surgeons and regional anesthesiologists.

**Funding for the Study:**

N/A